# Novel association of the obesity risk-allele near Fas Apoptotic Inhibitory Molecule 2 (*FAIM2*) gene with heart rate and study of its effects on myocardial infarction in diabetic participants of the PREDIMED trial

**DOI:** 10.1186/1475-2840-13-5

**Published:** 2014-01-06

**Authors:** Dolores Corella, Jose V Sorlí, José I González, Carolina Ortega, Montserrat Fitó, Monica Bulló, Miguel Angel Martínez-González, Emilio Ros, Fernando Arós, José Lapetra, Enrique Gómez-Gracia, Lluís Serra-Majem, Valentina Ruiz-Gutierrez, Miquel Fiol, Oscar Coltell, Ernest Vinyoles, Xavier Pintó, Amelia Martí, Carmen Saiz, José M Ordovás, Ramón Estruch

**Affiliations:** 1 Department of Preventive Medicine and Public Health, School of Medicine, University of Valencia, Valencia, Spain; 2 CIBER Fisiopatología de la Obesidad y Nutrición, Instituto de Salud Carlos III, Madrid, Spain; 3 Cardiovascula Risk and Nutrition Research Group, Institut Hospital del Mar d’Investigacions Mèdiques (IMIM), Barcelona, Spain; 4 Human Nutrition Unit, Faculty of Medicine, IISPV, University Rovira i Virgili, Reus, Spain; 5 Department of Preventive Medicine and Public Health, School of Medicine, University of Navarra, Pamplona, Spain; 6 Lipid Clinic, Endocrinology and Nutrition Service, Institut d’Investigacions Biomèdiques August Pi Sunyer (IDIBAPS), Hospital Clinic, Barcelona, Spain; 7 Department of Cardiology, Araba University Hospital, Vitoria, Spain; 8 Department of Family Medicine, Primary Care Division of Sevilla, San Pablo Health Center, Sevilla, Spain; 9 Department of Epidemiology, School of Medicine, University of Malaga, Malaga, Spain; 10 Department of Clinical Sciences, University of Las Palmas de Gran Canaria, Las Palmas de Gran Canaria, Spain; 11 Instituto de la Grasa, Consejo Superior de Investigaciones Científicas, Sevilla, Spain; 12 University Institute for Health Sciences Investigation, Hospital Son Dureta, Palma de Mallorca, Spain; 13 Department of Computer Languages and Systems, School of Technology and Experimental Sciences, Jaume I University, Castellón, Spain; 14 Primary Care Division, Catalan Institute of Health, Barcelona, Spain; 15 Lipids and Vascular Risk Unit, Internal Medicine, Hospital Universitario de Bellvitge, Hospitalet de Llobregat, Barcelona, Spain; 16 Department of Nutrition and Physiology, Faculty of Pharmacy, University of Navarra, Pamplona, Spain; 17 Department of Cardiovascular Epidemiology and Population Genetics, Centro Nacional de Investigaciones Cardiovasculares (CNIC), Madrid, Spain; 18 IMDEA Alimentación, Madrid, Spain; 19 Nutrition and Genomics Laboratory, JM-USDA Human Nutrition Research Center on Aging, Tufts University, Boston, MA, USA; 20 Department of Internal Medicine, Hospital Clinic, IDIBAPS, Barcelona, Spain; 21 Genetic and Molecular Epidemiology Unit, Valencia University, Blasco Ibañez, 15, 46010 Valencia, Spain

**Keywords:** Heart rate, FAIM2, Apoptosis

## Abstract

**Background:**

The Fas apoptotic pathway has been implicated in type 2 diabetes and cardiovascular disease. Although a polymorphism (rs7138803; G > A) near the Fas apoptotic inhibitory molecule 2 (*FAIM2*) locus has been related to obesity, its association with other cardiovascular risk factors and disease remains uncertain.

**Methods:**

We analyzed the association between the *FAIM2*-rs7138803 polymorphism and obesity, blood pressure and heart rate in 7,161 participants (48.3% with type 2 diabetes) in the PREDIMED study at baseline. We also explored gene-diet interactions with adherence to the Mediterranean diet (MedDiet) and examined the effects of the polymorphism on cardiovascular disease incidence per diabetes status after a median 4.8-year dietary intervention (MedDiet versus control group) follow-up.

**Results:**

We replicated the association between the *FAIM2*-rs7138803 polymorphism and greater obesity risk (OR: 1.08; 95% CI: 1.01-1.16; P = 0.011; per-A allele). Moreover, we detected novel associations of this polymorphism with higher diastolic blood pressure (DBP) and heart rate at baseline (B = 1.07; 95% CI: 0.97-1.28 bmp in AA vs G-carriers for the whole population), that remained statistically significant even after adjustment for body mass index (P = 0.012) and correction for multiple comparisons. This association was greater and statistically significant in type-2 diabetic subjects (B = 1.44: 95% CI: 0.23-2.56 bmp; P = 0.010 for AA versus G-carriers). Likewise, these findings were also observed longitudinally over 5-year follow-up. Nevertheless, we found no statistically significant gene-diet interactions with MedDiet for this trait. On analyzing myocardial infarction risk, we detected a nominally significant (P = 0.041) association in type-2 diabetic subjects (HR: 1.86; 95% CI:1.03-3.37 for AA versus G-carriers), although this association did not remain statistically significant following correction for multiple comparisons.

**Conclusions:**

We confirmed the *FAIM2*-rs7138803 relationship with obesity and identified novel and consistent associations with heart rate in particular in type 2 diabetic subjects. Furthermore, our results suggest a possible association of this polymorphism with higher myocardial infarction risk in type-2 diabetic subjects, although this result needs to be replicated as it could represent a false positive.

## Background

Obesity is a major determinant of type 2 diabetes. Over the last decade, multiple genome wide association studies (GWAs) have identified numerous polymorphisms associated with body mass index (BMI), and some of them have also been related to higher type 2 diabetes risk [1-7]. However, how the majority of these genes function in affecting anthropometric-related parameters is unknown. Moreover, both obesity and type 2 diabetes have been associated with higher blood pressure and cardiovascular disease risk [8,9]. Currently, to better understand the possible mechanisms involved, some studies have also analyzed the association between these obesity-related genes with cardiovascular risk factors such as hypertension, metabolic syndrome, etc. [10-13]. The *FAIM2* (Fas apoptotic inhibitory molecule 2) is one of the less well-known genes associated with obesity. The rs7138803 (G > A) single nucleotide polymorphism (SNP) located near the *FAIM2* locus was described for the first time to be associated with higher BMI in carriers of the minor allele by Thorleifsson et al. [4]. Later, Speliotes et al. [6] in a larger GWAs confirmed this association and estimated that the increase of BMI per variant allele would be ~ 0.12 kg/m^2^. However, the results of later studies in different populations have been diverse [14-20], suggesting some heterogeneity of the association depending on the characteristics of the population analyzed. *FAIM2,* also called *NMP35* (neural membrane protein 35), or *LFG* (lifeguard), is known to codify for an antiapoptotic protein, highly expressed in the brain that antagonizes the Fas pathway [21,22]. Fas (CD95 or APO-1) is a cell surface receptor, expressed in a variety of tissues. FasL is a member of the tumour necrosis factor superfamily and the natural ligand to Fas [23]. Various studies have associated both Fas and FasL concentrations with hypertension and cardiovascular risk [24,25], and there is a study in Chinese subjects showing an association between the *FAIM2*-rs7138803 polymorphism and diastolic blood pressure [26]. However, there are no published studies in humans that have analyzed the association between the *FAIM2*-rs7138803 polymorphism and resting heart rate, which is an independent and important risk factor of cardiovascular diseases [27-29], mainly in type 2 diabetic patients [30]. Although, in animal models, it has been shown that Faim2 is a novel neuroprotective molecule in the context of cerebral ischemia [31], there are no studies on humans that have analyzed the association between *FAIM2* polymorphisms and cerebrovascular disease risk. Only one previous study, carried out in a Chinese population [32], has examined the association between the FAIM2-rs7138803 polymorphism and cardiovascular disease, but no statistical association was found. Taking into account that the Fas apoptotic pathway has been implicated in type 2 diabetes and that a high glucose intake can induce vascular endothelial cell apoptosis and higher risk of myocardial infarction [33,34], we hypothesized that the association between the *FAIM2*-rs7138803 polymorphism and heart rate and cardiovascular disease would be greater in type 2 diabetic subjects. Additionally, diet may modulate this association, as one experimental study showing that nutritional state affects the expression of the Faim2 gene suggested [35]. Thus, our aims were: 1) To analyze the association of the *FAIM2*-rs7138803 polymorphism with anthropometrical measures, blood pressure and resting heart rate in the whole population and in type 2 diabetic subjects at baseline, also analyzing its modulation by adherence to the MedDiet; 2) To study the longitudinal association of the polymorphism with heart rate and with incidence of cardiovascular diseases (stroke and myocardial infarction) by diabetes status, also analyzing modulation by intervention with MedDiet.

## Methods

### Subjects

We analyzed 7,161 participants (3,049 men and 4,112 women) in the PREDIMED (PREvención con DIeta MEDiterránea) trial from whom the *FAIM2*-rs7138803 polymorphism was successfully determined. The PREDIMED study is a multi-center, randomized, controlled clinical trial (controlled-trials.com number, ISRCTN35739639) aimed at assessing the effects of the MedDiet on the primary prevention of cardiovascular disease [36]. The completion date of this study was December 2010 and the total number of randomized subjects was 7,447. The 7,161 participants included in the present study did not differ in the main characteristics from those of the total cohort. Additional file 1: Figure S1 shows the CONSORT flowchart of the trial. Details of the PREDIMED trial including sample size calculations and interim analysis have been fully described elsewhere [36-38]. Briefly, from October 2003 physicians in Primary Care Centers selected high cardiovascular risk subjects. Eligible subjects were community-dwelling people (55–80 years of age for men; 60–80 years of age for women) who fulfilled at least one of two criteria: type 2 diabetes [39] or three or more cardiovascular risk factors as previously detailed [36-38]. Exclusion criteria included a personal history of cardiovascular disease, any severe chronic illness, and drug or alcohol addiction [36]. Participants were randomly assigned to three interventions: MedDiet with extra-virgin olive oil, MedDiet with mixed nuts and control group (low-fat diet). Participants assigned to both MedDiet groups received intensive training to follow the MedDiet and allocations of either extra virgin olive oil (1 litre/week, for all the family) or mixed nuts (30 g/d) at no cost [36,37]. Participants assigned to the control diet received recommendations on reducing the intake of all types of fat. A detailed description of the nutritional interventions has been provided elsewhere [36]. The Institutional Review Board/Ethics Committee of each participating center approved the study protocol. All participants provided written informed consent. Participants were followed up for a median of 4.8 years (interquartile range, 2.8 to 5.8 years). Data were analyzed cross-sectionally (at baseline) as well as longitudinally (pooling together the MedDiet intervention groups versus the control group). By December 2010, a total of 502 participants (7.0%) had been lost to follow-up for 2 or more years. Dropout rates were higher in the control group (11%) than in the MedDiet groups (5%) (Additional file 1: Figure S1). As compared with participants who remained in the trial, those who dropped out were younger (by 1.4 years), had a higher BMI and a lower score for the adherence to the MedDiet as previously detailed elsewhere [36]. The effect of the *FAIM2*-rs7138803 polymorphism on heart rate was also examined longitudinally analyzing all subjects having heart rate data at baseline, at 1-year, at 3-years and at 5-years (n = 2,310). The reduction in the number of cases in the longitudinal analysis was mainly due no to patients being lost during follow up, but to the fact that patients were not all recruited in the same year at the beginning of the study, but progressively over the following years (from 2003 to 2009) as previously described [36]. Therefore, individuals who entered the study in 2003 completed the 5-year follow-up period in the year 2010, which is when follow-up ended, whereas those who entered the study in later years could not complete the 5-year follow-up period and so were not included in the longitudinal analyses of the study on heart rate. The Institutional Review Board/Ethics Committee of each participating center approved the study protocol. All participants provided written informed consent.

### Demographic, clinical, anthropometric and dietary measurements

The baseline examination included an assessment of standard cardiovascular risk factors, medication use, socio-demographic factors and lifestyle variables, as previously detailed [36,38]. Food consumption was determined by a validated semi-quantitative food frequency questionnaire [40]. Adherence to MedDiet at baseline was assessed by a validated 14-item questionnaire [41]. The final score ranged from 0 to 14. The greater the score obtained from the questionnaire, the greater the adherence to the MedDiet. Physical activity was estimated by the Minnesota Leisure-Time Physical Activity Questionnaire, as previously described [36].

Weight and height were measured with calibrated scales and a wall-mounted stadiometer, respectively. BMI was calculated as weight in kilograms divided by the square of height in meters.

At baseline and yearly thereafter, trained personnel measured blood pressure and heart rate in triplicate using a validated semiautomatic oscillometer (Omron HEM-705CP, Hoofddorp, the Netherlands) with a 5-minute interval between each measurement with the patients seated and at rest in a peaceful setting. The means of these measurements were calculated.

### Outcome ascertainment

The primary endpoint was the occurrence of the first major cardiovascular events and comprised myocardial infarction, stroke or cardiovascular death [36]. We used four sources of information to identify end-points: 1) repeated contacts with participants; 2) family physicians; 3) yearly review of medical records; and 4) consultation of the National Death Index. The end-point adjudication committee, whose members were blind to treatment allocation, examined all medical records related to end-points. Only end-points confirmed by the adjudication committee that occurred between October 1, 2003, and December 1, 2010 were included in the analyses. The criteria for adjudicating primary outcomes are detailed elsewhere [36].

### Biochemical determinations, DNA extraction and genotyping

At baseline, blood samples were obtained from each participant after an overnight fast and were frozen at -80°C. Fasting glucose, total cholesterol, triglycerides, HDL-C and LDL-C were measured using standard methods as previously described [37].

Genomic DNA was extracted from buffy-coat with the MagNaPure LC DNA Isolation Kit (Roche Diagnostics, Mannheim, Germany). The rs7138803 (G > A) polymorphism near *FAIM2* was genotyped on a 7900HT Sequence Detection System (Applied Biosystems, FosterCity, CA, USA) using a fluorescent allelic discrimination TaqMan™ assay. The calling rate was 98%. Genotype frequencies did not deviate from Hardy-Weinberg equilibrium expectations (P = 0.378).

### Statistical analyses

Data were analyzed both at baseline and longitudinally in the intervention trial. Chi-square tests were used to test differences in percentages. T and ANOVA tests were applied to compare crude means of continuous variables among genotypes at baseline. Multivariable adjustments for continuous variables were carried out by linear regression analysis and regression coefficients (B) were estimated. Models were sequentially adjusted for age, sex, center, type 2 diabetes, total energy intake, adherence to MedDiet, alcohol, tobacco, physical activity, hypertension, and medications (antihypertensive, lipid-lowering and hypoglycemic drugs) as indicated. Antihypertensive medication included angiotensin-converting enzymes (ACE) inhibitors (48% of the population took these), diuretics (21% of the population) and other antihypertensive drugs (beta blockers, calcium channel blockers, etc.) regardless of the dosage, as previously described [36]. Three dummy variables (taking or not taking the corresponding drug regardless of the dosage) were considered for the antihypertensive medication including ACE inhibitors, diuretics and other antihypertensive drugs. In addition a dummy variable (taking or not taking) for lipid-lowering drugs and two dummy variables (one for insulin and other for oral antidiabetic agents) were included in the adjustment for medications. Hypertension was defined as systolic blood pressure > =140 mm Hg or diastolic blood pressure > =90 mmHg or under antihypertensive medication. The *FAIM2*-rs7138803 polymorphism was first tested as additive for continuous traits analyzed. However, when the differences between heterozygous and homozygous subjects for the variant A-allele were very small, a recessive model was selected (for heart rate). A dichotomous variable for the pre-randomization adherence to MedDiet was created using the sample means as cut-off values (9 points). Obesity was defined as BMI > = 30 kg/m^2^. Logistic regression methods were also used to estimate the contribution of the *FAIM2*-rs7138803 polymorphism to predict obesity. In addition, BMI was categorized into four categories (BMI < 25 kg/m^2^; 25–30 kg/m^2^; 30–35 kg/m^2^ and > =35 kg/m^2^). Heart rate in beats per minute (bmp) was also categorized into five categories (<=60, 60–70, 70–80, 80–90 and > =90 bmp) for categorical analyses. The interaction between the *FAIM2*-rs7138803 polymorphism and adherence to the MedDiet in determining BMI, blood pressure and resting heart rate at baseline was tested in multivariable regression models (linear or logistic for categorical) including the corresponding main effects and interaction terms. Subgroup analyses stratified by type 2 diabetes status were carried out according to our “a priori” hypothesis and aim. The effect of the *FAIM2*-rs7138803 polymorphism on heart rate in the whole population and stratified by type 2 diabetes status was also examined longitudinally analyzing all subjects having heart rate data at baseline, at 1-year, at 3-years and at 5-years (n = 2,310) in a multivariable model of repeated measures with interactions terms after adjustment for the dietary intervention and the indicated covariates. Dietary intervention was considered as a dichotomous variable pooling together the MedDiet intervention groups *versus* the control group after having checked the homogeneity of the effect in the two MedDiet groups). In addition, we analyzed the interaction term between the *FAIM2*-rs7138803 and dietary intervention on changes in heart rate at 5-years by linear regression analysis with multivariable adjustment. Finally, we examined the association between the *FAIM*2-rs7138803 polymorphism and cardiovascular events (total cardiovascular events, myocardial infarction or stroke) in the whole population and in type 2 diabetic subjects by Cox regression models with the length of follow-up as the primary time variable and adjustment for the dietary intervention and the other covariates as indicated. The exposure time was calculated as the time between randomization and the date of a major cardiovascular event, the date when completing the last interview, December 1st 2010, or the date at death, whichever came first. Hazard ratios (HR) with 95% confidence intervals (CI) for the *FAIM2*-rs7138803 polymorphism (recessive model) for cardiovascular diseases were calculated in the population as a whole. In addition, we carried out the stratified analysis by type-2 diabetes status. Analyses were based on the intention-to-treat principle. In multivariable model 1 we adjusted for sex, age, center and dietary intervention. Multivariable model 2 included additional adjustments for BMI, total energy intake, alcohol, smoking, type-2 diabetes, medications and physical activity. Additional adjustments for baseline hypertension were also undertaken. The proportionality of hazards in determining the main outcomes in the PREDIMED study was tested with the use of time-varying covariates as previously described [36]. Here we graphically checked the assumption of proportional hazards for the genetic factor (*FAIM2*) by looking at the Log-Log plot of survival. We obtained parallel lines for the curves of genotypes in all the models fitted. Finally, Kaplan-Meier survival curves were plotted to estimate the probability of remaining free of myocardial infarction during follow-up. Statistical analyses were performed with the SPSS package, version 18.0 (SPSS, Chicago, IL). All tests were two-tailed and the nominal significance level was set at a P-value <0.05. In addition, the Bonferroni correction was applied to compensate for multiple comparisons. Our approach consisted of using an adjusted alpha level equal to the nominal alpha level (0.05), divided by the number of new hypothesis tested. With the aim of achieving a balance between type I and type II errors, we have chosen to undertake the Bonferroni correction considering the three new groups of variables that we were investigating (heart rate, blood pressure and cardiovascular diseases). Anthropometric measures were not included in the correction because in this case we were not dealing with a new hypothesis of association. Thus, the adjusted alpha was 0.05/3 = 0.017. The significance of each original (uncorrected) test was assessed at this level. Thus, a P-value <0.017 was considered to be statistically significant taking into account the correction for multiple comparisons.

## Results

Prevalence of the *FAIM2*-rs7138803 (G > A) genotypes was: 42.2% GG, 44.9%GA and 12.3% AA. Table 1 shows demographic, clinical, lifestyle and genetic characteristics of the 7,161 participants in the PREDIMED study according to the randomly assigned dietary intervention groups at baseline. We did not find statistically significant differences on analyzing demographic, lifestyle (dietary intake, smoking, drinking and physical activity), clinical (prevalence of type 2 diabetes, hypertension and medications) or biochemical parameters at baseline among the *FAIM2*-rs7138803 (Additional file 1: Table S1).

**Table 1:** Demographic, clinical, lifestyle and genetic characteristics of the study participants at baseline by intervention groups

	**Total**		**MedDiet with EVOO**		**MedDiet nuts**		**Control group**	
	**(n = 7,161)**		**(n = 2,469)**		**(n = 2,363)**		**(n = 2,329)**	
Age (years)	67.0	± 6.2	67.0	± 6.2	66.7	± 6.1	67.3	± 6.3
BMI (Kg/m^2^)	30.0	± 3.8	29.9	± 3.7	29.7	± 3.8	30.2	± 4.0
Waist circumference (cm)	100.4	± 10.6	100.2	± 10.4	100.2	± 10.5	100.8	± 10.8
Female sex: n,%	4112	(57.4)	1448	(58.6)	1271	(53.8)	1393	(59.8)
Systolic blood pressure (mmHg)	149.4	± 20.8	148.5	± 20.8	149.6	± 20.4	150.1	± 21.1
Diastolic blood pressure (mmHg)	83.4	± 11.0	83.1	± 10.9	83.7	± 10.9	83.3	± 11.1
Heart rate (bpm)	71.4	± 11.2	71.4	± 11.1	71.2	± 11.1	71.6	± 11.3
Current smokers: n,%	1003	(14.0)	345	(14.0)	339	(14.3)	319	(13.7)
Type 2 diabetes: n,%	3462	(48.3)	1238	(50.1)	1096	(46.8)	1128	(48.4)
Hypertension: n,%	5928	(82.8)	2026	(82.1)	1954	(82.7)	1948	(83.6)
Dyslipidemia: n,%	5172	(72.2)	1766	(71.5)	1730	(73.2)	1676	(72.0)
FAIM2-rs7138803 (G > A) : n,%								
GG	3019	(42.2)	1022	(41.4)	1000	(42.3)	997	(42.8)
GA	3235	(45.2)	1132	(45.8)	1062	(44.9)	1041	(44.7)
AA	907	(12.7)	315	(12.8)	301	(12.7)	291	(12.5)
Energy intake (kcal/d)	2274	± 606	2286	± 606	2317	± 610	2219	± 598
Total fat (% energy)	39.2	± 6.8	39.3	± 6.9	39.4	± 6.5	39.0	± 6.8
Saturated fat (% energy)	10.0	± 2.3	10.0	± 2.2	10.0	± 2.2	10.0	± 2.3
MUFA (% energy)	19.5	± 4.6	19.6	± 4.6	19.5	± 4.3	19.3	± 4.7
Proteins (% energy)	16.6	± 2.8	16.6	± 2.9	16.5	± 2.7	16.6	± 2.8
Carbohydrates (% energy)	41.9	± 7.1	41.7	± 7.2	41.5	± 7.0	42.3	± 7.2
Adherence to the MedDiet	8.6	± 2.0	8.7	± 2.0	8.7	± 2.0	8.4	± 2.1
Alcohol consumption (g/d)	8.4	± 14.2	8.6	± 14.4	9.2	± 15.0	7.5	± 13.1
Physical activity (MET-min/day)	231	± 239	232	± 231	247	± 246	215	± 241

Values are mean ± SD for continuous variables and number (%) for categorical variables. BMI indicates body mass index.

MUFA, Monounsaturated fatty acids; MedDiet, Mediterranean diet; EVOO, extra virgin olive oil.

### Association between the FAIM2-rs7138803 polymorphism anthropometrical variables, blood pressure and heart rate at baseline

We observed a statistically significant association between the *FAIM2*-rs7138803 polymorphism and BMI (B_adjusted_ = 0.15: 95% CI: 0.02-0.27 kg/m^2^ per variant-allele; P = 0.023) in the additive model after adjustment for sex, age, centre, type 2 diabetes, hypertension and medications (Table 2). We also found additive effects in the association of this polymorphism with obesity (OR_adjusted:_ 1.08; 95% CI: 1.01-1.17 per variant-allele; P: 0.011). Likewise, we obtained a statistically significant association with diastolic blood pressure (DBP) in the additive model adjusted for covariates that remained significant after BMI adjustment (P = 0.015). Moreover, we observed a novel association between this polymorphism and resting heart rate which was greater in the recessive model, and that remained statistically significant after multivariable adjustment including BMI (P = 0.012). Additional adjustment of the model for DBP did not attenuate the statistical significance of this association (P = 0.010). All these association were statistically significant not only at the nominal P-value (P < 0.05) but also after correction for multiple comparisons taking the level of P < 0.017 into account.

**Table 2:** **Association between the *FAIM2*-rs7138803 and anthropometrical measures, blood pressure and heart rate**

			***FAIM2 *** **(G > A) genotypes**											
	**GG (n = 3,019)**		**GA (n = 3,235)**		**AA (n = 907)**		**Additive model**				**Recessive model**			
	**Mean**	**SD**	**Mean**	**SD**	**Mean**	**SD**	**P**^**1**^	**B**^**1 **^**(95% CI)**	**P**^**2**^	**P**^**3**^	**B**^**1 **^**(95% CI)**	**P**^**1**^	**P**^**2**^	**P**^**3**^
Weight (kg)	76.4	± 11.9	76.9	± 11.9	77.3	± 12.1	0.026	0.46 (0.06-0.87)	0.059		0.59 (-0.24-1.42)	0.164	0.191	
BMI (kg/m2)	29.9	± 3.8	30.0	± 3.9	30.1	± 3.9	0.055	0.13 (-0.01-0.26)	0.023		0.20 (-0.07-0.47)	0.147	0.066	
Waist circumference (cm)	100.3	± 10.5	100.3	± 10.6	101.0	± 10.9	0.167	0.26 (-0.11-0.62)	0.141		0.68 (-0.07-1.42)	0.075	0.085	
SBP (mmHg)	149.2	± 20.8	149.4	± 20.9	149.9	± 20.1	0.414	0.30 (-0.42-1.01)	0.783	0.889	0.57 (-0.89-2.03)	0.443	0.761	0.896
DBP (mmHg)	82.9	± 10.9	83.4	± 10.9	84.3	± 11.2	0.001	0.61 (0.24-1.00)	0.006	0.015	1.12 (0.36-1.89)	0.004	0.009	0.017
Heart rate (bpm)	71.1	± 11.2	71.2	± 10.9	72.3	± 11.4	0.029	0.43 (0.04-0.81)	0.039	0.048	1.07 (0.29-1.86)	0.007	0.010	0.012
Prevalence of obesity (%)	45.2		47.5		49.1		0.021					0.136		
Obesity risk, OR (95% CI)	1 (ref.)		1.10 (0.99-1.21)		1.17 (1.01-1.35)		0.021	1.08 (1.01-1.16)	0.011		1.11 (0.97-1.28)	0.136	0.070	

Means, prevalence, odds ratios (OR), and regression coefficients (B).

Values are means and standard deviations (SD), odds ratio (OR) and 95% confidence intervals (CI) or regression coefficients (B) and 95% CI.

B: Regression coefficient per-variant allele effects in the additive model (genotypes coded as 0, 1 and 2 according to the number of minor alleles for additive effects) and regression coefficients for homozygotes for the variant allele versus the other genotypes in the recessive model (genotypes coded as 0 for GG and GA and 1 for AA).

SBP: Systolic blood pressure. DBP: Diastolic blood pressure.

^1^: Unadjusted P values for the polymorphism for the comparison of means or OR (in an additive or recessive models as indicated).

^2^: Models adjusted for sex, age, center, type 2 diabetes, medications (antihypertensive drugs, lipid-lowering drugs and antidiabetic drugs) and diagnosed hypertension.

^3^: Model 2 additionally adjusted for BMI. Further adjustment for total energy intake, adherence to Mediterranean diet and physical activity did not change the statistical significance of these associations.

In the stratified analysis by type-2 diabetes status (48% diabetic patients), we observed that the association of the polymorphism with heart rate was greater and statistically significant (P = 0.010) in type-2 diabetic subjects (B_adjusted_ = 1.44: 95% CI: 0.23-2.56 bmp for AA versus carriers of the G-allele). No significant association was found in non-diabetics (B_adjusted_: 0.62: 95% CI: -0.47-1.69 bmp; P = 0.258, in non-diabetics for AA versus G-carriers). However, in terms of BMI, we found no heterogeneity of the effects of the polymorphism by diabetes status, obtaining similar associations in type 2 diabetic subjects as in non-diabetics (not shown).

We also analyzed (Additional file 1: Table S2) the association of the *FAIM2*-rs7138803 polymorphism with different categories of BMI and observed that the association increased with BMI categories (P-for trend: 0.008). Categorizing heart rate (Table 3) into five categories (<=60, 60–70, 70–80, 80–90 and > =90 bpm), we also observed that the association with the polymorphism was greater as the heart rate category increased (P-for linear trend: 0.002). Thus, prevalence of AA homozygotes was 9.9% in subjects having a heart rate < =60 bpm, whereas its prevalence was 15.0% among subjects having a heart rate > =90 bpm (OR_adjusted_: 1.66, 95% CI: 1.13-2.43; P = 0.009 for AA homozygotes versus G-allele carriers) in the multivariable adjusted model including BMI.

**Table 3:** **Association between the *FAIM2*-rs7138803 polymorphism and categories of heart rate**

	***FAIM2 *** **(G > A) genotypes**			**Unadjusted**^**1**^		**Adjusted**^**2**^		**Adjusted**^**3**^	
**Heart rate**	**GG + GA (n = 6,049)**		**AA (n = 888)**	OR* (95% CI)	**P**^**1**^	OR* (95% CI)	**P**^**2**^	OR* (95% CI)	**P**^**3**^
<60 bpm (%)	90.1		9.9	1 (ref)		1 (ref)		1 (ref)	
60-70 bmp (%)	87.3		12.7	1.32 (1.05-1.67)	0.019	1.27 (1.00-1.60)	0.051	1.26 (1.00-1.60)	0.052
70-80 bmp (%)	86.4		13.6	1.43 (1.13-1.82)	0.003	1.46 (1.14-1.87)	0.003	1.45 (1.13-1.86)	0.003
80-90 bmp (%)	86.1		13.9	1.46 (1.12-1.92)	0.006	1.47 (1.10-1.97)	0.009	1.46 (1.10-1.96)	0.011
> = 90 bmp (%)	85.0		15.0	1.60 (1.13-2.27)	0.008	1.65 (1.13-2.42)	0.010	1.66 (1.13-2.43)	0.009
P for trend*		0.002							

Prevalence and odds ratios (OR). Unadjusted and adjusted models.

Values are prevalences in%, odds ratio (OR) and 95% confidence intervals (CI).

OR: Odds ratio for the corresponding category of heart rate in comparison with the reference category (<60 bpm) for homozygous subjects for the variant allele (recessive model) in comparison with carriers of the major allele.

*: P-value of the Chi^2^ test for linear trend.

^1^: Unadjusted P values for the polymorphism (recessive model).

^2^: Models adjusted for sex, age, center, type 2 diabetes, hypertension, physical activity, medications, smoking, drinking, adherence to Mediterranean diet and total energy intake.

^3^: Model 2 additionally adjusted for BMI.

### Modulation by adherence to the MedDiet at baseline of the associations between the FAIM2-rs7138803 polymorphism and anthropometrical variables, DBP and heart rate

We did not observe any statistically significant gene-diet interaction in the multivariable adjusted models (by sex, age, centre, type 2 diabetes, medications, hypertension, smoking, drinking, physical activity and total energy intake) between adherence to the MedDiet (two categories based on the population mean of 9 points) at baseline and the *FAIM2*-rs7138803 polymorphism in determining BMI (P-interaction: 0.716) (Additional file 1: Figure S2A), waist circumference (P-interaction: 0.658), obesity (P-interaction = 0.679), DBP (P-interaction: 0.346) or heart rate (P-interaction: 0.371) (Additional file 1: Figure S2B).

### Stratified analysis per hypertension of the association between the FAIM2-rs7138803 polymorphism and DBP and heart rate

Although in this study, prevalence of diagnosed hypertension at baseline was very high (82.8%), we analyzed whether the association of the polymorphism with DBP (Additional file 1: Figure S3A) and heart rate (Additional file 1: Figure S3B) differed by hypertension status. We obtained no statistically significant interactions and observed homogeneous results across the strata. Statistically significant differences for the polymorphism were detected in subjects with hypertension because this group had a higher sample size.

### Longitudinal association between the FAIM2-rs7138803 polymorphism and heart rate

Given that that heart rate was the parameter in which we found the most novel association, we wanted to study whether the association between the *FAIM2*-rs7138803 polymorphism and heart rate was maintained over time and how the intervention with MedDiet (MedDiet groups *vs* control group after having observed no heterogeneity in the MedDiet groups) influenced that association. We used longitudinal data from 5-year follow-up analyzing data for all subjects having heart rate measured at baseline, at 1-year, at 3-years and at 5-years (n = 2,310) in a model for repeated measures. Prevalence of the *FAIM2*-rs7138803 genotypes in this group was 41.3% GG, 44.8% GA and 13.9% AA, very close to the population as a whole. The mean age was the same (67.1 ± 6.0 years) as in the population as a whole. There were slight differences in other demographic or clinical variables. Thus, in this group prevalence of women was 56.4%, prevalence of type 2 diabetes was 45.1%, mean BMI was 29.7 ± 3.6 kg/m^2^, mean SBP was 84.4 ± 10.8 mmHg and mean heart rate was 70.5 ± 10.8 bpm.

Having firstly tested an additive model and observed similar effects in heterozygous and in homozygous subjects, we employed the recessive model. For the whole population, we detected a statistically significant effect of the polymorphism in determining the heart rate over the 5-year follow-up period (P = 0.004 in the model adjusted for intervention with MedDiet, sex, age, centre, baseline BMI, type 2 diabetes, hypertension, medications, smoking and physical activity). We did not obtain a significant interaction between the *FAIM2*-rs7138803 polymorphism and intervention with MedDiet in the follow-up for the whole population (P-interaction: 0.392, in the adjusted model). Neither did we obtain significant interactions between these variables in determining changes in heart rate (P = 0.215).

**Figure 1:**
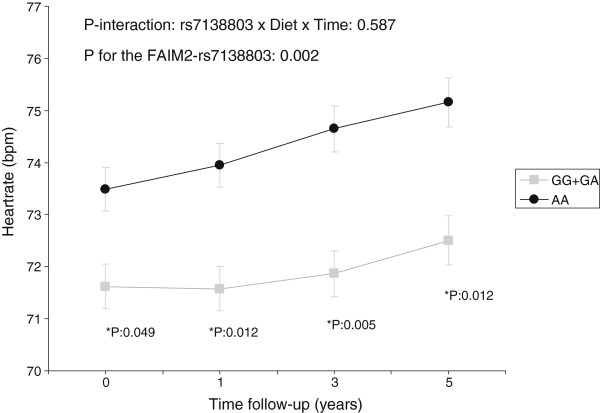
**Longitudinal effect of the *FAIM2*-rs7138803 polymorphism on heart rate in type 2 diabetic subjects.** Adjusted means of heart rate in beats per minute (bpm) depending on the polymorphism (GG + GA versus AA) at baseline, 1, 3 and 5-years of follow-up in all type 2 diabetic subjects at baseline having data for all four measurements (n = 1,076). Adjusted means were estimated from a repeated-measures ANOVA model with interaction terms adjusted for dietary intervention (MedDiet *versus* control), sex, age, BMI, medications, smoking, and physical activity. Adjusted P values for the interaction term among the polymorphism, time, and dietary intervention as well for the overall effect of the polymorphism are shown. *Adjusted P values for the polymorphism at every specific time point. Further adjustment of the model for hypertension at baseline did not change the statistical significance of the results.

In the analysis stratified by diabetes status (n = 1,076 subjects with type-2 diabetes and n = 1,234 non-diabetics), we found that the longitudinal association between the *FAIM2*-rs7138803 polymorphism and heart rate was higher and statistically significant in subjects with type-2 diabetes (P = 0.002 for the polymorphism in the longitudinal analysis of the multivariable adjusted model) (Figure 1). This longitudinal association remained statistically significant after correction for multiple comparisons. No statistically significant association for the polymorphism in the follow-up was detected in non-diabetics (P = 0.135). Even in type 2 diabetic subjects we did not detect a significant interaction between the polymorphism and the intervention with MedDiet (P-interaction = 0.587, multivariable adjusted) in the follow-up.

### Association between FAIM2-rs7138803 polymorphism and incidence of cardiovascular events and mortality after 4.8-years follow-up

**Figure 2:**
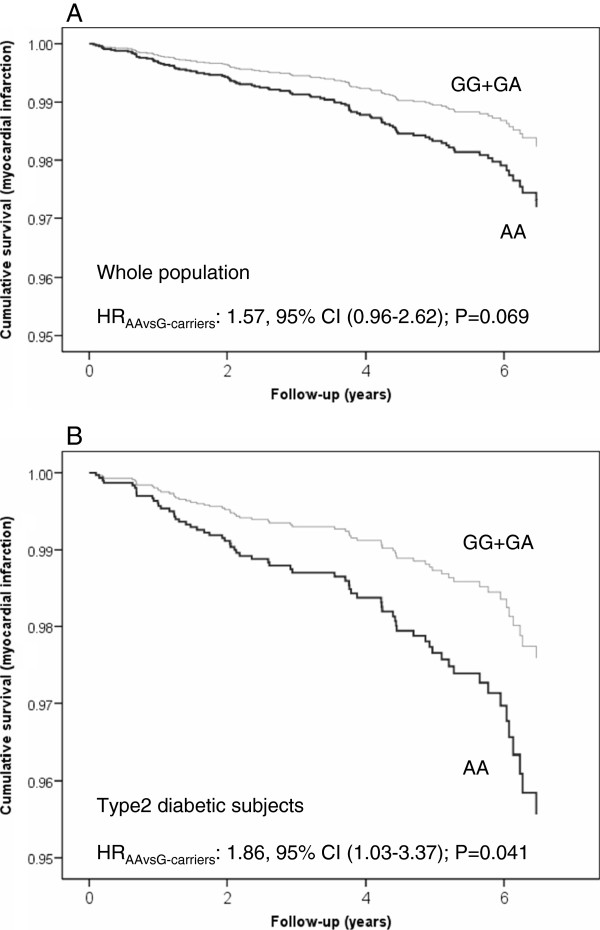
**Cumulative myocardial infarction free-survival by the *FAIM2*-rs7138803 polymorphism in the whole population (A) (n = 7,161) and in type 2 diabetic subjects (B) (n = 3,462).** Cox regression models with outcome of myocardial infarction and the rs7138803 polymorphism (G-allele carriers *versus* AA) adjusted by dietary intervention group, sex, age, centre, type 2 diabetes, hypertension, medications, BMI, total energy intake, alcohol, smoking and physical activity. The P-values for the *FAIM2*-rs7138803 polymorphism were obtained from the multivariable adjusted models.

After a median follow-up of 4.8 years (interquartile range, 2.8 to 5.8 years), 268 major cardiovascular events occurred among the 7,161 participants analyzed (30,360 person-years of observation). Of these, 135 were strokes, 100 myocardial infarctions and the others were cardiovascular deaths. Firstly, we longitudinally analyzed the association of the polymorphism with the incidence of cardiovascular events and adjusted the model for dietary intervention (MedDiets and control group). Additional file 1: Table S3 of the Supporting Online Material shows the HRs for total cardiovascular events, stroke and myocardial infarction. In the whole population, we did not observe a significant association between the *FAIM2*-rs7138803 polymorphism (in the recessive model) and myocardial infarction either in the basic model or in the model adjusted for BMI (HR_adjusted_: 1.57, 95% CI: 0.96-2.62). In the analysis stratified by type 2 diabetes, we observed a nominally significant association in type 2 diabetes patients (HR_adjusted_:1.86; 95% CI:1.03-3.37; P = 0.041 in AA versus G-carriers). However, this association did not remain statistically significant following correction for multiple comparisons. Figure 2 shows cumulative myocardial infarction free-survival by the *FAIM2*-rs7138803 polymorphism in the whole population (**A**) and in type-2 diabetic subjects (**B**).

In the study of the modulation by the MedDiet (MedDiet versus control group) of the effects of the *FAIM2*-rs7138803 polymorphism on the incidence of cardiovascular events, we did not find significant results. None of the interaction terms between MedDiet intervention and the polymorphism reached the nominal statistical significance (not shown). Although, we have sample size limitations to detect gene-diet interaction terms as statistically significant, this lack of significance seems to be derived from homogeneous results. Thus, in the case of myocardial infarction, a similar trend to a greater risk was observed in AA subjects compared with G-carriers both in the control group (HR: 1.66, 95% CI: 0.70-3.81) as in the MedDiet intervention groups (HR: 1.59; 95% CI: 0.85-2.99).

## Discussion

In this study on high cardiovascular risk subjects (48% patients with type 2 diabetes) from a Mediterranean population, we found, as in other studies [4,6,8,18,20,42], that the variant allele of the *FAIM2*-rs7138803 polymorphism was associated with higher obesity risk. Furthermore, our results support the observations of Paternoster et al. [7] and Sandholt et al. [20] who found that this polymorphism was associated with higher degrees of obesity (BMI > 35). Although it is known that the *FAIM2* gene codifies an evolutionary conserved inhibitor of Fas-mediated apoptosis [21-23] and the apoptotic pathways are higher in obesity [43,44], the mechanisms by which this association occurs are not known. As we have found for the first time, a strong association between this polymorphism and resting heart rate, as well as with DBP, we can hypothesize on the influence of the *FAIM2*-rs7138803 polymorphism. Thus, the minor allele may give rise to a variant resulting in less *FAIM2* gene expression or activity. Hence minor allele carriers seem to be less protected against apoptosis and appear to present higher levels (despite their magnitude being small) of harmful cardiovascular risk phenotypes (greater obesity, higher DBP and higher resting heart rate), contributing over time increasing the potential risk of myocardial infarction. Although the functions of the FAIM2 in the different metabolic diseases are not well known, it is known that the FAIM2 acts as an antiapoptotic protein which would inhibit Fas-mediated cell death [45]. Moreover a growing body of evidence has shown that Fas-mediated apoptosis is involved in atherosclerosis progression [25,45,46]. A study in rats [47] suggested that the augmented expression of apoptotic signalling by the Fas/FasL pathway plays an important role in hypertensive nephrosclerosis. In humans it has also been reported that hypertensive subjects present a greater expression of the FAS gene [48]. In addition, greater serum concentrations of both Fas and FasL have been associated with type-2 diabetes, hypertension, and cardiovascular disease [24,33,34,45,49,50]. In our study, we have described for the first time a statistically significant association between the *FAIM2*-rs7138803 polymorphism and higher DBP, which was independent from BMI and remained significant even after correction for multiple comparisons. Although in a previous study on Chinese subjects [26] the *FAIM2*-rs7138803 polymorphism was associated with higher DBP in children, this association did not remain statistically significant after adjustment for BMI. Moreover, as far as we know, no previous study has reported an association between the *FAIM2*-rs7138803 polymorphism and heart rate, this association being greater and statistically significant in type 2 diabetic subjects. Moreover, we not only observed this association at baseline but also after analyzing the association of this polymorphism with heart rate longitudinally. The fact that this novel association was found consistently during the 5 years of follow-up in a sub-group of individuals provides greater consistency to this association.

On the other hand, when we studied the modulation of these effects by the MedDiet we did not observe any statistically significant interaction either in the study at baseline or following intervention with MedDiet over 4.8 years follow-up. Future studies are therefore required to better characterize the possible interactions with more specific dietary components. Numerous studies have reported that increased heart rate is a predictor of cardiovascular events and specifically of myocardial infarction [29,51-54]. In our study, we observed that the polymorphism was nominally (P < 0.041) associated with higher risk of myocardial infarction in type 2 diabetes subjects. This would be in line with previous studies that have reported higher apoptosis in type 2 diabetic subjects [33,34,55], greater association between increased heart rate and cardiovascular morbid-mortality in type-2 diabetes subjects [29,30,56] and with studies showing that apoptosis in cardiomyocytes is a very important risk factor in triggering myocardial infarction [57,58]. However, in our study this association did not remain statistically significant after correction for multiple comparisons. This correction is a hotly debated issue in biomedical literature, many authors being opposed to such corrections because of the increase in false negative results. Only one previous study [32], has examined the association between the *FAIM2*-rs7138803 polymorphism and cardiovascular disease, and no statistical association was found. There are many differences between our study and the Chinese population analyzed and further research is needed into this issue. Another of the polymorphisms associated with obesity in recent GWAs (the rs4923461 polymorphism in the *BNDF* gene) has been associated with higher cardiovascular risk regardless of BMI [59], so providing new evidence of the pleiotropic effects of obesity-related polymorphisms.

### Limitations

Our study has several limitations, one of them being the relatively low number of cardiovascular events (myocardial infarction and stroke) that reduces the statistical power for detecting statistically significant associations between the *FAIM2* polymorphism and cardiovascular events as well as to detect gene-diet interactions between the polymorphism and MedDiet intervention in determining cardiovascular events. In line with this observation, we may also have statistical power limitations to detect gene-diet interaction in determining heart rate in the follow-up because only a sub-group of subjects had data at 5-y. At baseline, our sample size consisting of 7,161 subjects was high and allowed us to detect as statistically significant differences in heart rate or DBP around unit. These associations although statistically significant were small in magnitude and may be clinically non-relevant.

## Conclusions

In conclusion, the results of our study confirm the association between the *FAIM2*-rs7138803 polymorphism and obesity risk, being greater for higher degrees of obesity. Moreover we provide new data on the association between this polymorphism and higher DBP and heart rate regardless of BMI, these associations being higher in subjects with type 2 diabetes. In addition, the association of the polymorphism with heart rate was consistently found over the follow-up, adding more evidence to this novel association. All of this may contribute to the fact that homozygous subjects for the variant allele tended to have a higher risk of myocardial infarction than the other genotypes in type 2 diabetic subjects. This association, although nominally significant did not pass the correction for multiple comparisons and needs to be replicated in further studies.

## Competing interests

ER is a non paid member of the Scientific Advisory Committee of the California Walnut Commission, Sacramento, CA. The other authors have no competing interest affecting the conduct or reporting of the work submitted.

## Authors’ contributions

DC, RE, JMO, JVS, MAMG, ER, JL and LSM designed research; COA, JVS, JIG, MAMG, EGG, MB, MF, OC, ER, RE, FA, CS, EV, and LSM conducted research; JVS, MAMG, MB, MF, RE, FA, JL, LSM, EGG, MF, AM, VRG, XP, and EV provided essential materials; DC, COA, JVS, OC and JMO analyzed data and performed statistical analysis; DC, JVS, and JMO wrote paper; DC and JVS had primary responsibility for final content. All authors made substantial contributions to conception and design, acquisition of data or analysis and interpretation of data, drafting the article or revising it critically for important intellectual content and approved the final version of the manuscript.

## Supplementary Material

Additional file 1: Figure S1 Flow-chart of the trial. **Figure S2.** Interaction between the FAIM2-rs7138803 polymorphism and adherence to the MedDiet in determining BMI (A) and heart rate (B). **Figure S3.** Stratified analysis of the association between the FAIM2-rs7138803 and diastolic blood pressure (DBP) (A) and heart rate (B) depending on hypertension. **Table S1.** Demographic, lifestyle, clinical and biochemical characteristics of the participants depending on the *FAIM2*-rs7138803 polymorphism. **Table S2.** Association between the *FAIM2*-rs7138803 polymorphism and categories of obesity. Prevalence and odds ratios (OR). Unadjusted and adjusted models. **Table S3.** Association between the *FAIM2*-rs7138803 polymorphism and incidence of cardiovascular diseases in the whole population and by type 2 diabetes status. Hazard ratios (HR) and 95% confidence intervals (CI). Click here for file
