# Supplementary material for: Novel association of the obesity risk-allele near Fas Apoptotic Inhibitory Molecule 2 (FAIM2) gene with heart rate and study of its effects on myocardial infarction in diabetic participants of the PREDIMED trial
Source: Cardiovasc Diabetol. 2014 Jan 6;13:5. doi: 10.1186/1475-2840-13-5 (PMC3922966; doi:10.1186/1475-2840-13-5)
Supplement: Additional file 1: Figure S1 — Flow-chart of the trial. Figure S2. Interaction between the FAIM2-rs7138803 polymorphism and adherence to the MedDiet in determining BMI (A) and heart rate (B). Figure S3. Stratified analysis of the association between the FAIM2-rs7138803 and diastolic blood pressure (DBP) (A) and heart rate (B) depending on hypertension. Table S1. Demographic, lifestyle, clinical and biochemical characteristics of the participants depending on the FAIM2-rs7138803 polymorphism. Table S2. Association between the FAIM2-rs7138803 polymorphism and categories of obesity. Prevalence and odds ratios (OR). Unadjusted and adjusted models. Table S3. Association between the FAIM2-rs7138803 polymorphism and incidence of cardiovascular diseases in the whole population and by type 2 diabetes status. Hazard ratios (HR) and 95% confidence intervals (CI). [file 1475-2840-13-5-S1.pdf]

SUPORTING MATERIAL

Figure S1: Flow-chart of the participants

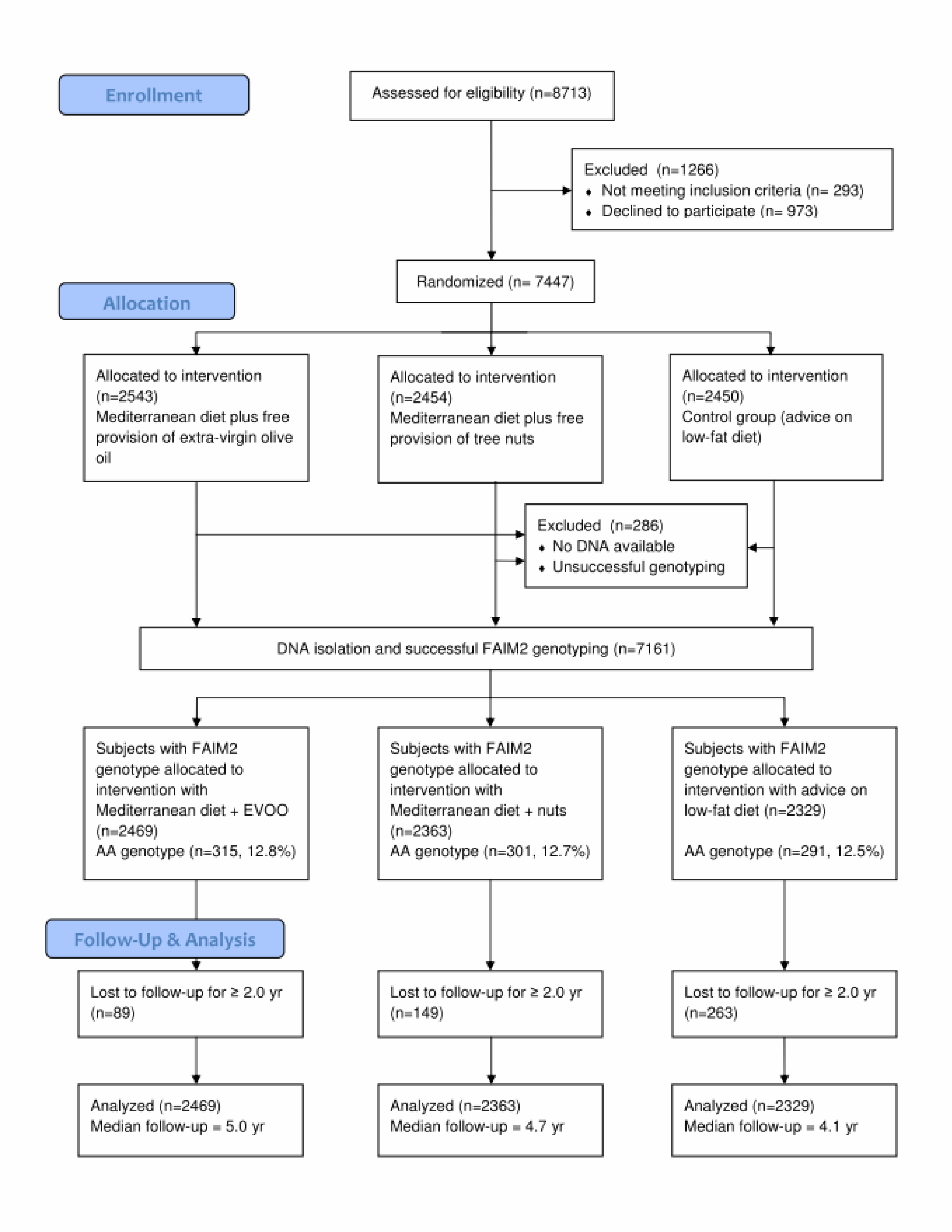

Figure S2. **Interaction between the FAIM2-rs7138803 polymorphism and adherence to the MedDiet in determining BMI (A) and heart rate (B)**.
Adjusted means depending on the strata of adherence to the Mediterranean diet (MedDiet) and the *FAIM2*-rs7138803. MedDiet was considered as dichotomous (low <9 points and high ≥ 9 points). Means were adjusted for sex, age, centre, type 2 diabetes, medications, smoking, drinking, physical activity and total energy intake. The model for the polymorphism was additive for BMI (n=7,161) and recessive for heart rate (n=6,937). P-values for the interaction terms were multivariable adjusted. Error bars: SE of means.

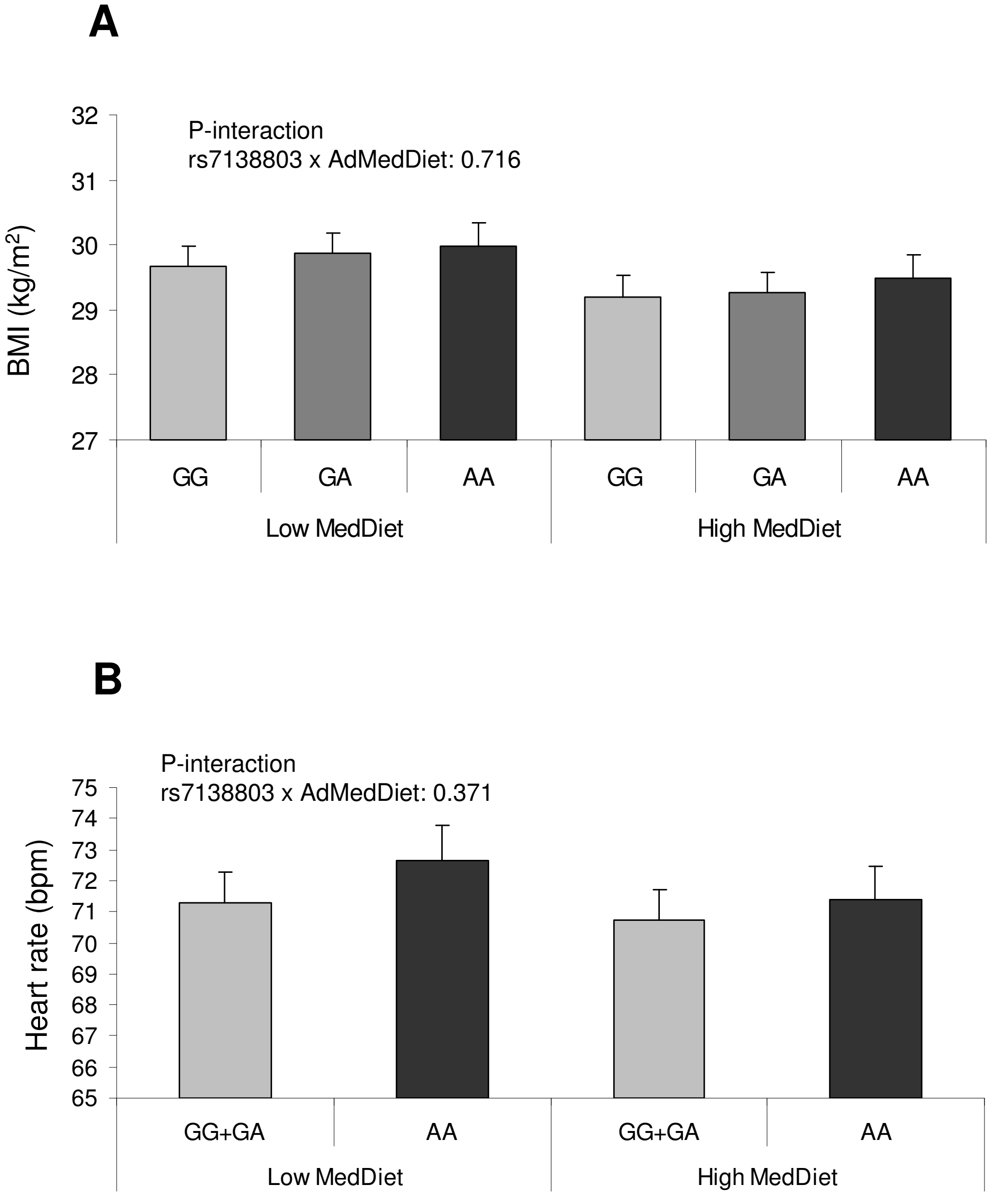

Figure S3. Stratified analysis of the association between the *FAIM2*-rs7138803 and diastolic blood pressure (DBP) (A) and heart rate (B) depending on hypertension. Adjusted means depending on the hypertensive status diagnosed at baseline (yes or not) and the FAIM2-rs7138803. Means were adjusted for sex, age, centre, type 2 diabetes, medications, smoking, drinking, physical activity and total energy intake. The model for the polymorphism was additive for DBP (n=7,129) and recessive for heart rate (n=6,937). P-values for the interaction terms were multivariable adjusted. Multivariable adjusted P-values for the polymorphism in determining DBP were 0.089 and 0.036 in non-hypertensive subjects and hypertensive subjects, respectively. P-values for the polymorphism in determining heart in the same strata rate were 0.401 and 0.016, respectively. *P<0.017. Error bars: SE of means.

A

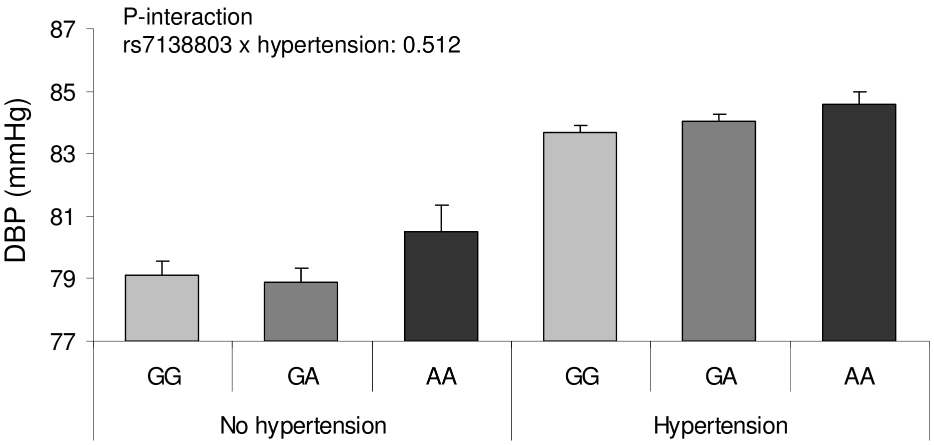

B

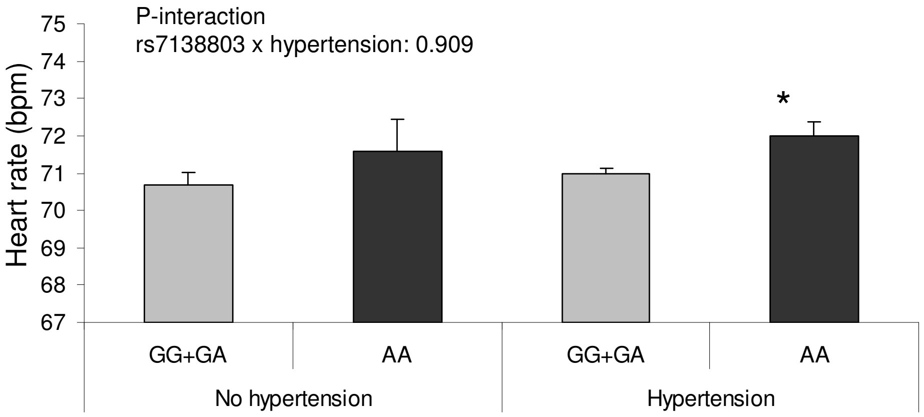

Supplemental Table 1: Demographic, lifestyle, clinical and biochemical characteristics of the participants depending on the *FAIM2*-rs7138803 polymorphism

	GG (n=3,019)	GA (n=3,235)	AA (n=907)	
Parameter	Mean SD	Mean SD	Mean SD	P[1]
Age (years)	67.0 ± 6.2	66.9 (6.2)	66.9 (6.3)	0.528
Energy intake (kcal/d)	2274 ± 605	2276 ± 604	2275 ± 601	0.988
Total fat (% energy)	39.2 ± 6.8	39.2 ± 6.8	39.1 ± 6.5	0.963
Saturated fat (% energy)	10.0 ± 2.3	10.0 ± 2.2	10.0 ± 2.2	0.901
MUFA (% energy)	19.4 ± 4.6	19.4 ± 4.6	19.4 ± 4.3	0.511
Proteins (% energy)	16.6 ± 2.8	16.5 ± 2.8	16.6 ± 2.8	0.608
Carbohydrates (% energy)	41.9 ± 7.2	41.8 ± 7.2	41.9 ± 6.9	0.939
Adherence to the MedDiet	8.6 ± 2.0	8.7 ± 2.0	8.6 ± 2.0	0.447
Alcohol consumption (g/d)	8.4 ± 14.3	8.5 ± 14.1	8.2 ± 14.1	0.790
Physical activity (MET-min/day)	231 ± 232	235 ± 254	224 ± 211	0.447
Fasting glucose^ (mg/dL)	121.0 ± 41.4	122.6 ± 41.5	122.9 ± 42.1	0.294
Total cholesterol^ (mg/dL)	111.6 ± 38.4	110.8 ± 40.4	110.2 ± 38.2	0.575
LDL-C^ (mg/dL)	130.8 ± 34.1	130.0 ± 35.9	129.7 ± 34.7	0.576
HDL-C^ (mg/dL)	54.3 ± 14.5	53.4 ± 13.9	53.7 ± 13.3	0.068
Triglycerides^ (mg/dL)	135.0 ± 75.9	139.2 ± 82.2	137.9 ± 76.3	0.133
Female sex : n, %	1761 (58.3)	1847 (57.1)	504 (55.6)	0.296
Type 2 diabetes: n, %	1436 (47.6)	1570 (48.5)	546 (50.3)	0.344
Hypertension: n, %	2492 (82.5)	2687 (83.1)	749 (84.6)	0.851
Dyslipidemia: n, %	2180 (72.2)	2346 (72.5)	646 (71.2)	0.743
Antihypertensive drugs: n, %	2195 (72.9)	2354 (72.8)	657 (72.4)	0.703
Lipid lowering drugs: n, %	1427 (47.3)	1581 (48.9)	441 (48.6)	0.354
Oral antidiabetic drugs: n;%	937 (31.0)	1026 (31.7)	322 (35.5)	0.141
Insulin: n, %	197 (6.5)	234 (7.2)	58 (6.4)	0.631
Current smokers: n, %	428 (14.2)	439 (13.6)	136 (15.0)	0.851

*: Values are means and standard deviations (SD) or n (%) or odds ratio, OR and 95% confidence intervals (95% CI)

[1]: Unadjusted P values for mean comparison among genotypes

total energy intake, alcohol consumption, smoking and physical activity.

^: Fasting glucose, total cholesterol, LDL-C, HDL-C and triglycerides were obtained for n= 6317, n=6694, n=6612, n=6588, n=6622 subjects, respectively

Supplemental Table 2: Association between the *FAIM2*-rs7138803 polymorphism and categories of obesity. Prevalence and odds ratios (OR). Unadjusted and adjusted models

	***FAIM2* (G>A) Genotypes**			Unadjusted[1]		Adjusted[2]	
	GG (n=3,019)	GA (n=3,235)	AA (n=907)	**OR* (95%CI)**	**P[1]**	**OR* (95%CI)**	**P[2]**
BMI<25 kg/m^2, (%)	45.8	42.1	12.1	1 (ref)		1 (ref)	
BMI: 25-30 kg/m^2, (%)	42.9	44.9	12.1	1.07 (0.93-1.22)	0.359	1.08 (0.94-1.23)	0.303
BMI: 30-35 kg/m^2, (%)	41.0	46.0	13.0	1.13 (0.99-1.29)	0.082	1.13 (0.99-1.30)	0.077
BMI >=35 kg/m^2, (%)	39.9	45.4	14.6	1.19 (1.01-1.40)	0.043	1.20 (1.01-1.44)	0.046
P for trend		0.008					

Values are prevalences in %, odds ratio (OR) and 95% confidence intervals (CI)

OR: Odds ratio for the corresponding category of BMI in comparison with the reference category (BMI<25 kg/m2) for each variant allele (additive model)

[1]: Unadjusted P values for the polymorphism (additive model)

[2]: Models adjusted for sex, age, center, type 2 diabetes, drinking, smoking, physical activity, adherence to Mediterranean diet and total energy intake.
Further adjustment for hypertension did not change the statistical significance of results

Supplemental Table 3. Association between the *FAIM2*-rs7138803 polymorphism and incidence of cardiovascular diseases in the whole population and by type 2 diabetes status. Hazard ratios (HR) and 95% confidence intervals (CI)

	**Whole population**	**Type 2 diabetes status**	
	(n=7,161)	Type 2 diabetics (n=3,462)	No-diabetics (n=3,699)
	HR 95% CI	HR 95% CI	HR 95% CI
Total cardiovascular events*			
FAIM2 genotypes (Model 1)			
G carriers	1.00 (ref.)	1.00 (ref.)	1.00 (ref.)
AA	1.18 (0.84-1.65)	1.08 (0.71-1.63)	1.35 (0.77-2.34)
	P^1=0.344	P^1=0.727	P^1=0.293
FAIM2 genotypes (Model 2)			
G carriers	1.00 (ref.)	1.00 (ref.)	1.00 (ref.)
AA	1.17 (0.83-1.62)	1.07 (0.79, 1.64)	1.34 (0.77-2.32)
	P^2=0.399	P^2=0.758	P^2=0.303
Myocardial Infarction			
FAIM2 genotypes (Model 1)			
G carriers	1.00 (ref.)	1.00 (ref.)	1.00 (ref.)
AA	1.60 (0.97-2.64)	1.83 (1.01-3.09)	1.21 (0.47-3.11)
	P^1=0.065	P^1=0.046	P^1=0.697
FAIM2 genotypes (Model 2)			
G carriers	1.00 (ref.)	1.00 (ref.)	1.00 (ref.)
AA	1.59 (0.97-2.63)	1.86 (1.03-3.37)	1.20 (0.46-3.09)
	P^2=0.068	P^2=0.041	P^2=0.710
Stroke			
FAIM2 genotypes (Model 1)			
G carriers	1.00 (ref.)	1.00 (ref.)	1.00 (ref.)
AA	1.01 (0.61-1.65)	0.89 (0.47-1.70)	1.30 (0.57, 2.85)
	P^1=0.981	P^1=0.680	P^1=0.563
FAIM2 genotypes (Model 2)			
G carriers	1.00 (ref.)	1.00 (ref.)	1.00 (ref.)
AA	0.98 (0.60-1.61)	0.87 (0.46-1.64)	1.24 (0.56-2.85)
	P^2=0.934	P^2=0.668	P^2=0.597

*Composite end point including myocardial infarction, stroke and cardiovascular mortality

Model 1: Adjusted for sex, age, center and dietary intervention groups

Model 2: Adjusted for sex, age, center, dietary intervention groups, BMI, medications, type 2 diabetes, alcohol, smoking, total energy intake and physical activity

P^1: P value obtained for the genotype effect (recessive model) of the polymorphism in the multivariable adjusted model 1. Cox regression models in the whole population or by type 2 diabetes

P^2: P value obtained for the genotype effect (recessive model) of the polymorphism in the multivariable adjusted model 2. Cox regression models in the whole population or by type 2 diabetes

Additional adjustment of the models for hypertension (Model 3), did not change the statistical significance of the results (P^3: 0.069, 0.041 and 0.718 for the association between the FAIM2 genotype and myocardial infarction in the whole population, in type 2 diabetic patients and in non-diabetic subjects, respectively)
